# Determinants and Within-Person Variability of Urinary Cadmium Concentrations among Women in Northern California

**DOI:** 10.1289/ehp.1205524

**Published:** 2013-04-03

**Authors:** Robert B. Gunier, Pamela L. Horn-Ross, Alison J. Canchola, Christine N. Duffy, Peggy Reynolds, Andrew Hertz, Erika Garcia, Rudolph P. Rull

**Affiliations:** 1 Cancer Prevention Institute of California, Berkeley, California, USA; 2 School of Public Health, University of California, Berkeley, Berkeley, California, USA; 3 Department of Health Research and Policy, Stanford University School of Medicine, Stanford, California, USA; 4 School of Community Health Sciences, University of Nevada, Reno, Reno, Nevada, USA

**Keywords:** cadmium, biomarkers, diet, exposure science, GIS

## Abstract

Background: Cadmium (Cd) is a toxic metal associated with increased morbidity and mortality. Urinary Cd (U-Cd) concentration is considered a biomarker of long-term exposure.

Objectives: Our objectives were to evaluate the within-person correlation among repeat samples and to identify predictors of U-Cd concentrations.

Methods: U-Cd concentrations (micrograms per liter) were measured in 24-hr urine samples collected from 296 women enrolled in the California Teachers Study in 2000 and a second 24-hr sample collected 3–9 months later from 141 of the participants. Lifestyle and sociodemographic characteristics were obtained via questionnaires. The Total Diet Study database was used to quantify dietary cadmium intake based on a food frequency questionnaire. We estimated environmental cadmium emissions near participants’ residences using a geographic information system.

Results: The geometric mean U-Cd concentration was 0.27 µg/L and the range was 0.1–3.6 µg/L. The intraclass correlation among repeat samples from an individual was 0.50. The use of a single 24-hr urine specimen to characterize Cd exposure in a case–control study would result in an observed odds ratio of 1.4 for a true odds ratio of 2.0. U-Cd concentration increased with creatinine, age, and lifetime pack-years of smoking among ever smokers or lifetime intensity-years of passive smoking among nonsmokers, whereas it decreased with greater alcohol consumption and number of previous pregnancies. These factors explained 42–44% of the variability in U-Cd concentrations.

Conclusion: U-Cd levels varied with several individual characteristics, and a single measurement of U-Cd in a 24-hr sample did not accurately reflect medium- to long-term body burden.

Cadmium (Cd) is a toxic, bioaccumulating, and somewhat persistent metal released into the environment during mining operations and industrial processes and as a by-product of oil combustion [Agency for Toxic Substances and Disease Registry (ATSDR) 1999]. Nonoccupational Cd exposure, assessed using urinary Cd (U-Cd) levels from a single sample, has been associated with kidney disease (Järup and Åkesson 2009; Suwazono et al. 2006), cardiovascular disease (Peters et al. 2010), dental caries (Arora et al. 2008), decreases in bone mineral density (Satarug and Moore 2004), and increased mortality (Menke et al. 2009; Nawrot et al. 2008). Cd is classified as a human lung carcinogen (International Agency for Research on Cancer 1993) and has been associated with increased overall cancer mortality (Adams et al. 2012) and the incidence of breast (Gallagher et al. 2010; Julin et al. 2012; McElroy et al. 2006) and endometrial cancers (Åkesson et al. 2008).

Cd is stored in the liver and kidneys, has a biological half-life of 10–30 years, and is absorbed via inhalation and ingestion (ATSDR 1999). Absorption of Cd in the gastrointestinal tract is limited (3–10%), whereas absorption from the deep areas of the lung is high (50–90%), suggesting that inhalation may be an important route of exposure (Waalkes 2003). The principal source of exposure for smokers in nonindustrial settings is inhalation of cigarette smoke [Centers for Disease Control and Prevention (CDC) 2005], and smoking may double the daily intake of Cd compared with not smoking (ATSDR 1999). For nonsmokers, the principal source of Cd is ingestion of contaminated plant-based foods (CDC 2005). Women generally have higher internal Cd levels than men because depleted iron stores and iron deficiency, common among women of childbearing age, increase the intestinal uptake of Cd (Vahter et al. 2002).

Numerous studies have cited U-Cd concentration as a reliable measure of cumulative lifetime exposure (Julin et al. 2011; McElroy et al. 2006; Nawrot et al. 2006). Accurate assessment of long-term Cd exposure is important because U-Cd levels have been associated with health outcomes thought to have a long latency period such as cancer and cardiovascular disease. However, short-term Cd exposure levels could also be important for studies of prenatal exposure and developmental effects in children. U-Cd concentrations have been shown to be correlated with age (Hellstrom et al. 2004), sex (Hellstrom et al. 2004), iron deficiency (Berglund et al. 1994), parity (Åkesson et al. 2002), smoking status (Hellstrom et al. 2004; Ikeda et al. 2005), secondhand smoke (Willers et al. 2005), and dietary intake of Cd (Adams et al. 2011; Choudhury et al. 2001; Julin et al. 2011; Shimbo et al. 2000). However, most of these exposure studies have relied on single spot urine samples instead of repeated 24-hr urine collections. In addition, sources of Cd exposure among nonoccupationally exposed and mostly nonsmoking women have not been well characterized. The objective of this study was to identify determinants of U-Cd using repeat 24-hr urine collections and exposure information from a variety of sources including self-reports, environmental databases, and a dietary contaminant database in a sample of women enrolled in the largely nonsmoking California Teachers Study (CTS) cohort.

## Methods

*Study population and questionnaire data*. Our study population consisted of 296 women participating in a measurement substudy of the CTS cohort. The cohort includes 133,479 women who were active or retired public school teachers or administrators in 1995 (Bernstein et al. 2002). The substudy, conducted in 2000, included a random sample of CTS participants who resided in the substudy area (i.e., western Alameda, Santa Clara, San Mateo, Santa Cruz, Monterey, or northern San Benito counties in California) and were ≤ 85 years of age at baseline in 1995–1996 (Gunier et al. 2006; Horn-Ross et al. 2008). Of the 484 women invited to participate in the substudy, 328 (68%) agreed, 138 refused, and 18 were not interviewed for other reasons. All participants provided written informed consent and this research was approved by the institutional review board of the Cancer Prevention Institute of California. Of the 328 participants, 304 (93%) provided a 24-hr urine specimen in 2000. Our analysis is based on 296 of these women who had adequate urine volume available for Cd analysis. Of the 157 women asked, 141 (90%) provided a second 24-hr urine specimen; these samples were collected 3, 6, or 9 months after the initial sample. Both the original and repeat specimens were analyzed for U-Cd.

Information on parity, duration of breastfeeding, and active smoking history was obtained from self-administered questionnaires completed when the CTS cohort was established in 1995–1996, and information on the source, setting (household, workplace, and social), timing, and dose of passive smoking exposures was obtained from a second survey mailed to CTS participants in 1997. For nonsmokers, we used a measure of lifetime intensity-years of passive smoking calculated by multiplying a qualitative description of smoke intensity (1 = a little smoky, 2 = fairly smoky, or 3 = very smoky) by duration of exposure in years (Reynolds et al. 2009). Current age, usual diet, and alcohol consumption during the past year, weight, height, and current residential address were obtained from substudy participants at the time of urine sample collection in 2000.

*Sample collection and laboratory analysis*. Each substudy participant received a collection kit and was instructed to collect all urine produced in the 24-hr period starting immediately following the in-person interview. The samples were collected and stored at –20°C for ≤ 2 weeks until they were thawed, aliquotted, and frozen at –70°C. Approximately 9 years elapsed between the sample collection in 2000 and the analysis of Cd concentrations.

U-Cd concentrations (micrograms per liter) were measured using inductively coupled plasma–mass spectrometry at a certified commercial laboratory (Pacific Toxicology Laboratories, Chatsworth, CA). The limit of detection (LOD) for U-Cd was 0.1 µg/L. Low and high Cd control standards were included in each batch to evaluate assay accuracy and precision. The within-batch coefficient of variation was < 10% and between-batch coefficient was < 15%. Creatinine concentrations (grams per liter) were measured using a modified-rate Jaffe method and were highly correlated with creatinine concentrations measured in 2000 at another laboratory [intraclass correlation coefficient (ICC) = 0.88].

*Environmental and dietary exposure assessment*. We estimated potential environmental exposure to Cd at the substudy participants’ geocoded residences in 2000 using a geographic information system (GIS) and three available databases for industrial emissions, ambient air concentrations, and vehicle traffic. To estimate exposure to Cd emissions from industrial and commercial facilities, we used 1995 data from the California Air Toxics Emissions Data System, which provides latitude and longitude coordinates and annual emissions in pounds self-reported by each facility (California Air Resources Board 1998). We estimated the distance between a residence and all facilities within 5 km with reported Cd emissions. Geocoded residences were also linked by census tract to estimated Cd concentrations in ambient air in 1999 from the National Air Toxics Assessment (U.S. Environmental Protection Agency 2006). These concentrations were derived using an atmospheric dispersion model that combined emissions inventories with local meteorology (Rosenbaum et al. 1999).

To estimate potential exposure to Cd from vehicle emissions, we obtained traffic count data for 2000 from the California Highway Performance and Monitoring System (California Department of Transportation 2007). These data provide the annual average daily traffic (AADT), the average number of vehicles per day traveling in both directions on major roads. For each participant’s residence, we calculated traffic density by summing the vehicle kilometers of travel (VKT) within a 300-m radius buffer by multiplying the AADT by the length of the road segment for each road segment with AADT values within the buffer, then dividing by the buffered area (0.28 km^2^) to obtain VKT per day per square kilometer (Gunier et al. 2003). We used a 300-m radius because this approximates the distance at which particulate pollutant concentrations approach background levels (Zhou and Levy 2007).

Dietary Cd intake was assessed via an early version of the 103-item Block95 food frequency questionnaire (FFQ) administered to substudy participants in 2000 (Block et al. 1986, 1990; Horn-Ross et al. 2008). For each food item, frequency of consumption (categories ranging from never to once per day or ≤ 5 times per day, depending on the item), and usual portion size (small, medium, or large relative to a given standard medium portion) were assessed for the previous year (i.e., 1999). The FFQs were self-administered and checked by study staff for completeness. FFQ items were assigned Cd values based on the Total Diet Study market basket surveys conducted between 1991 and 2004 [Food and Drug Administration (FDA) 2006]. Dietary Cd was not estimated for three participants who did not complete the FFQ and six participants whose reported food consumption was judged to be implausibly low (< 600 calories/day) or high (> 5,000 calories/day).

*Statistical analysis*. For seven samples with U-Cd concentration below the LOD, we assigned a concentration equivalent to the LOD divided by the square root of 2 (specifically, 0.07 µg/L). We calculated the creatinine-adjusted U-Cd levels (micrograms per gram creatinine) by dividing the U-Cd concentrations (micrograms per liter) by the creatinine concentrations (grams per liter). We multiplied U-Cd concentration (micrograms per liter) by the total volume of urine collected during the 24-hr period (liters per day) to estimate daily Cd output (micrograms per day). Potential explanatory variables for the variation in U-Cd concentrations included age at the time of urine sample collection (2000) rescaled so that the youngest person had an age of zero years, body mass index (BMI; kilograms per meter squared, a measure of weight independent of height), and body surface area [(weight (in kilograms)^0.425^ × height (in centimeters)^0.275^ × 0.007184), a measure of body size reflecting muscle mass] (Ruggieri and Rocca 2010) using height and weight from 2000, parity (i.e., still and live births were combined to obtain the total number of full-term pregnancies), total duration of breastfeeding (months), oral contraceptive use (ever/never), and hormone replacement therapy (ever/never) as of 1995–1996; lifetime active and passive smoking history (through 1997); usual alcohol consumption (grams per day), dietary Cd intake (micrograms per day), and environmental indicators of potential exposure from traffic, industrial, and commercial sources as of 1999. Because the distribution of U-Cd was skewed, we used the nonparametric Kruskal–Wallis test to make categorical comparisons of the U-Cd distribution from the first urine sample collected from each participant (*n* = 296) and demographic, dietary, and environmental characteristics. We tested continuous and ordinal predictor variables for a linear trend in regression models using natural logarithm–transformed U-Cd concentrations as the dependent variable to normalize the U-Cd distribution and adjusting for age at urine collection because age was a strong predictor of U-Cd levels.

Variance components models with random intercepts for each participant were used to determine the ICC of U-Cd concentrations from repeated samples collected from the same individual. We calculated the ratio of the within- and between-person variance (λ = variance ratio) and the attenuation bias that would result from measurement error in a case–control study using a single measure of U-Cd to estimate exposure where the expected value of the logistic regression coefficient for U-Cd (β_E_) is related to the true regression coefficient (β_T_), the variance ratio (λ), and the number of measurements per person (*n*) by the equation β_E_⊇=⊇β_T_/(1+λ/*n*). We calculated the attenuation bias in a case–control study resulting from exposure misclassification as the normalized difference between the expected value and true value of the logistic regression coefficient using percent attenuation bias (% attenuation bias)⊇=⊇(β_E_–β_T_)/β_T_ × 100 (Whitehead et al. 2012).

We used linear mixed-effects models with random intercepts to identify significant predictors (*p* < 0.1) of U-Cd levels and to estimate the amount of variability in measured levels explained by the model while accounting for the correlation among repeat samples collected from the same individual (Peretz et al. 2002). We included creatinine concentration as a predictor in our models with unadjusted U-Cd levels as the dependent variable instead of using creatinine-adjusted U-Cd as the dependent variable because this allows for an evaluation of the relationship between U-Cd and other predictor variables independent of urinary creatinine concentration (Barr et al. 2005). We first identified potential explanatory variables from questionnaire data that were associated with U-Cd concentrations with *p* < 0.2 in either bivariate or linear trend analyses. We then used a backward stepwise elimination regression to derive final multivariate models including all questionnaire variables that predicted U-Cd concentrations with *p* < 0.1, in addition to the environmental and dietary Cd exposure estimates that were retained in final models regardless of their *p*-values. To estimate associations with passive tobacco smoke exposure, we created a separate model restricted to women who never smoked (*n* = 163). We used information on the years since former smokers (*n* = 70) quit smoking by adding 3 years to data collected in 1997 to estimate the percentage decrease in U-Cd concentrations per year after smoking cessation adjusted for other significant predictors. We performed a 10-fold cross-validation to evaluate the fit of our models by setting aside 10% of the data and rerunning the models (Shao 1993). All analyses were performed using SAS, version 9.2 (SAS Institute Inc., Cary, NC) and Stata, version 11 (StataCorp, College Station, TX).

## Results

Table 1 presents the characteristics of the study participants. At enrollment into the CTS, substudy participants had a median of two full-term pregnancies, had breastfed for a median duration of 3 months, and most had never smoked (68%). At the time of urine collection in 2000, substudy participants were on average 55 years of age, had a slightly greater than ideal body mass (median⊇=⊇25 kg/m^2^), and had an average body surface area of 1.8 m^2^. Traffic density (0–427,000 VKT/km^2^) and industrial Cd emissions (0–1,760 kg) at participant residences in 2000 ranged over several orders of magnitude, and interquartile ranges for estimated dietary Cd intake and Cd concentrations in ambient air were 7.9–14 µg/day and 0.09–0.28 ng/m^3^, respectively. The geometric means (GMs) of U-Cd concentration, creatinine-adjusted U-Cd concentration, and 24-hr U-Cd output from the first 24-hr urine sample were 0.27 µg/L, 0.38 µg/g, and 0.46 µg/day, respectively.

**Table 1:** Distributions of demographic, lifestyle, and geographic factors and laboratory data from the first 24-hr urine samples provided by participants

Variable	*n*	Minimum	Percentile			Maximum	Mean±SD
			25th	50th	75th		
Self-reported data
Age (years)	296	31	47	54	62	84	55±12
BMI (kg/m^2^)	293	16	23	25	29	61	27±5.9
Body surface area (m^2^)	293	1.2	1.7	1.8	1.9	2.7	1.8±0.2
Parity (full-term pregnancies)	291	0	0	2	3	6	1.7±1.4
Breastfeeding (months)	290	0	1	3	5	9	3.2±2.3
Smoking (pack-years)	296	0	0	0	1.0	61	3.9±10
Passive smoking (intensity-years)^*a*^	171	0	4.0	21	40	203	30±35
Dietary Cd intake (µg/day)^*b*^	287	2.5	7.9	11	14	28	11±4.2
Geographic exposure data
Industrial emissions (kg)^*c*^	296	0	0	0	0.003	1,760	18±146
Outdoor air (ng/m^3^)	296	0.05	0.09	0.15	0.28	0.65	0.20±0.13
Traffic density (VKT/km^2^)^*d*^	296	0	0	7,000	33,900	427,000	27,500±55,200
Urinary concentrations
Unadjusted Cd (µg/L)	296	0.1	0.2	0.3	0.4	2.0	0.27 (1.9)^*e *^
Creatinine (g/L)	296	0.1	0.5	0.7	1.0	2.5	0.71 (1.6)^*e *^
Creatinine-adjusted Cd (µg/g)	296	0.1	0.3	0.4	0.5	1.5	0.38 (1.8)^*e *^
Cd output (µg/day)	295	0.1	0.3	0.5	0.7	2.6	0.46 (1.7)^*e *^
^***a***^Among never-smokers responding to a 1997 questionnaire about exposure to secondhand smoke (*n*=171), calculated by multiplying duration of exposure in years by intensity (1,a little smoky; 2,fairly smoky; 3,very smoky).^***b***^Estimated from food frequency questionnaire combined with cadmium levels measured in food items from the Total Diet Study (FDA 2006).^***c***^Cd emissions within 5km of residence.^***d***^Traffic density, VKT/km^2^ within 300 m of residence.^***e***^GM (geometric SD).							

The ICC among the 141 participants with repeated 24-hr urine samples was 0.50 for U-Cd concentration and 0.42 for creatinine-adjusted U-Cd concentration, indicating moderate within-person correlation over time. The ICC was similar whether the time between repeat urine sample collection was 3, 6, or 9 months (ICC = 0.51, 0.59, and 0.42 respectively). Based on the overall within- and between-person variance components (0.221 and 0.216, respectively; a variance ratio of 1.0), measurement error resulting from the use of a single 24-hr U-Cd sample to estimate exposure would result in a 50% attenuation bias of the regression coefficient toward the null, whereas the use of two or four U-Cd samples would result in 33% and 20% attenuation bias respectively.

Table 2 presents selected results from analyses of self-reported characteristics and U-Cd levels in the first 24-hr urine sample (*n* = 296). U-Cd levels increased significantly (*p* < 0.1) with both age and cumulative pack-years of smoking, and the relationship was stronger for creatinine-adjusted U-Cd concentrations (*p* < 0.0001). Passive smoking was not related to U-Cd concentrations using either categorical or continuous measures. The GM creatinine-adjusted U-Cd concentration among those with ≥ 20 pack-years of smoking (0.57 µg/g) was 63% higher than the GM levels among never-smokers (0.35 µg/g). There was a significant linear trend (*p* = 0.0001) of decreasing U-Cd concentrations as category of average daily alcohol consumption increased from none to < 20 g/day (one drink) to ≥ 20 g/day (two or more drinks). Increasing parity was also related to lower U-Cd levels (*p* =⊇0.02) and was a stronger predictor of creatinine-adjusted U-Cd levels (*p* =⊇0.0002). Larger body surface area was associated with lower unadjusted (*p* =⊇0.04) and creatinine-adjusted U-Cd (*p* = 0.08), whereas higher BMI was associated with lower creatinine-adjusted U-Cd (*p* = 0.08) but not unadjusted U-Cd concentrations (*p* =⊇0.14). Duration of breastfeeding (*p* = 0.05) and ever use of hormone replacement therapy (*p* = 0.02) were associated with creatinine-adjusted U-Cd but not unadjusted U-Cd. Never using oral contraceptives was associated with higher unadjusted (*p* = 0.002) and creatinine-adjusted U-Cd concentrations (*p* = 0.001). There was no relationship between U-Cd or creatinine-adjusted U-Cd levels and estimated dietary Cd intake. Although U-Cd levels increased with categorical estimated exposure to all three environmental sources of Cd, these differences were not significant nor was there a significant linear trend with the exception of industrial Cd emissions within 5 km and unadjusted U-Cd concentrations (*p* = 0.06) (Table 3).

**Table 2:** Selected participant and lifestyle characteristics and 24-hr U-Cd concentrations from the first sample provided, unadjusted and adjusted for creatinine.

Characteristic	*n *(%)	Unadjusted Cd (μg/L)			Creatinine-adjusted Cd (μg/g)		
		GM (μg/L)	Kruskal–Wallis *p*-value^*a*^	Linear trend *p*-value^*b*^	GM (μg/g)	Kruskal–Wallis *p*-value^*a*^	Linear trend *p*-value^*b*^
Age (years)
31–39	30 (10)	0.26	0.11	0.01	0.30	<0.0001	<0.0001
40–49	63 (21)	0.23			0.31		
50–59	114 (39)	0.27			0.38		
60–84	89 (30)	0.30			0.45		
Smoking (pack-years)
0 (never)	207 (70)	0.26	0.02	0.01	0.35	0.0002	<0.0001
0.1–4.9	40 (13)	0.25			0.40		
5.0–19.9	26 (9)	0.27			0.43		
≥ 20	23 (8)	0.41			0.57		
Passive smoking (intensity-years)^*c *^
<4	42 (25)	0.26	0.15	0.70	0.34	0.27	0.53
4–20	44 (27)	0.31			0.40		
21–40	43 (25)	0.24			0.34		
>40	37 (23)	0.28			0.37		
Alcohol (g/day)
None	96 (32)	0.32	0.0005	0.0001	0.44	0.0006	0.0001
<20	174 (59)	0.25			0.36		
≥ 20	26 (9)	0.19			0.29		
Parity (full-term pregnancies)
0	75 (26)	0.28	0.65	0.02	0.41	0.39	0.0002
1–2	143 (49)	0.27			0.37		
3	43 (15)	0.24			0.35		
>3	30 (10)	0.25			0.37		
Total duration of breastfeeding (months)
≤ 1	81 (27)	0.28	0.37	0.17	0.41	0.03	0.05
2–3	81 (27)	0.29			0.40		
4–5	87 (29)	0.25			0.33		
>5	47 (16)	0.25			0.36		
Oral contraceptive use
Ever	201 (72)	0.25	0.002	—	0.35	0.001	—
Never	80 (28)	0.32			0.44		
Hormone replacement therapy
Ever	141 (48)	0.27	0.31	—	0.38	0.02	—
Never	154 (52)	0.27			0.37		
BMI (kg/m^2^)
<25.0	145 (50)	0.28	0.30	0.14	0.40	0.15	0.08
25.0–29.9	82 (28)	0.24			0.35		
≥ 30.0	66 (22)	0.28			0.36		
Body surface area (m^2^)
<1.65	73 (25)	0.31	0.04	—	0.42	0.08	—
≥ 1.65	223 (75)	0.25			0.36		
^***a***^Non­parametric test using the Kruskal–Wallis one-way analysis of variance by ranks.^***b***^Linear test for trend from regression model of natural logarithm-transformed concentrations adjusted for age.^***c***^Among never-smokers responding to the 1997 questionnaire about exposure to secondhand smoke (*n*=171), calculated by multiplying duration of exposure in years by intensity (1,a little smoky; 2,fairly smoky; 3,very smoky).							

**Table 3:** Dietary and environmental characteristics and 24-hr U-Cd concentrations from the first sample provided, unadjusted and adjusted for creatinine.

Characteristic	*n *(%)	Unadjusted Cd (μg/L)			Creatinine adjusted Cd (μg/g)		
		GM (μg/L)	Kruskal–Wallis *p*-value^*a*^	Linear trend *p*-value^*b*^	GM (μg/g)	Kruskal–Wallis *p*-value^*a*^	Linear trend >*p*-value^*b*^
Dietary cadmium intake (μg/day)
<7.9	71 (25)	0.28	0.72	0.30	0.38	0.82	0.98
7.9–10.6	72 (25)	0.27			0.37		
10.6–13.7	73 (25)	0.27			0.37		
>13.7	71 (25)	0.25			0.38		
Estimated outdoor cadmium concentration (ng/m^3^)
<0.1	94 (32)	0.25	0.37	0.29	0.36	0.34	0.31
0.1–0.3	142 (48)	0.27			0.38		
>0.3	60 (20)	0.29			0.39		
Traffic density (VKT/km^2^)
0	101 (34)	0.27	0.78	0.82	0.37	0.64	0.54
1–7,000	48 (16)	0.27			0.36		
7,001–70,000	118 (40)	0.26			0.38		
>70,000	29 (10)	0.29			0.41		
Industrial cadmium emissions (kg within 5 km)
0	203 (69)	0.26	0.23	0.06	0.37	0.57	0.23
0.001–20	52 (18)	0.27			0.38		
> 20	41 (14)	0.30			0.40		
^***a***^Non­parametric test using the Kruskal–Wallis one-way analysis of variance by ranks.^***b***^Linear test for trend from regression model of natural logarithm-transformed concentrations adjusted for age.							

Table 4 provides the estimated percentage change in U-Cd concentrations from the final mixed-effects models based on all available 24-hr urine samples with smoking as a predictor variable for all participants (model 1) and among never-smokers with passive smoking intensity-years as a predictor variable (model 2). The total variance explained [coefficient of determination (*R*^2^)] was similar at 42–44% for both models. The greatest variability in U-Cd concentrations was explained by creatinine concentration (27%) and age (8%). Total pack-years of smoking among all participants and total lifetime intensity of passive smoking among nonsmokers were also positively associated with U-Cd. Each year in age was associated with a 1.4% increase (95% CI: 0.9, 1.9) in U-Cd concentration and each pack year of active smoking was associated with a 1% increase (95% CI: 0.5, 1.6). Among former smokers (*n* = 70 participants and 97 samples), the number of years since smoking stopped was associated (*p* = 0.01) with a 1.5% decrease (95% CI: –2.7, –0.3) per year in U-Cd concentration (data not shown). Increasing parity and alcohol intake were associated with lower U-Cd concentrations. Dietary and environmental estimates of Cd exposure were not significant predictors (*p* > 0.1) of U-Cd concentrations in this population. Body surface area, BMI, breastfeeding duration, use of hormone replacement therapy and oral contraceptives were dropped from the final multivariate models because they were not significant predictors (*p* > 0.1).

**Table 4:** Estimated adjusted percentage change (95% CI) in 24-hr U-Cd concentration (µg/L) associated with potential predictors.

Variable (categories, if applicable)	Model1 (all participants)			Model2 (never-smokers)		
	Percentchange^*a*^ (95% CI)	*p*-Value	*R*^2^	Percentchange^*a*^ (95% CI)	*p*-Value	*R*^2^
No. of samples/subjects	412/285			233/161		
Creatinine (per 0.1 g/L)	15 (12, 19)	<0.001	0.27	14 (11, 19)	<0.001	0.27
Age (per year)^*b*^	1.4 (0.9, 1.9)	<0.001	0.35	1.1 (0.40, 1.8)	0.003	0.35
Smoking (per lifetimepack-year)^*c*^	1.1 (0.5, 1.6)	0.001	0.37	Not included		
Passive smoking (per lifetime intensity-year)^*d*^	Not included			0.2 (0.0, 0.4)	0.05	0.37
Total full-term pregnancies (perpregnancy)^*e*^	–4.6 (–8.6, –0.5)	0.004	0.39	–5.2 (–10.1, 0.0)	0.05	0.39
Alcohol intake (0, <20, ≥20 g/day)^*b*^	–16 (–23, –7.4)	<0.001	0.40	–16 (–24, –4.9)	0.01	0.40
Cadmium in air (per 0.1ng/m^3^)^*b*^	–1.4 (–7.9, 5.5)	0.77	0.40	1.0 (–7.4, 11)	0.28	0.41
Industrial emissions—within 5 km (per 10-fold change inkg)^*b*^	2.5 (–1.9, 7.1)	0.27	0.41	1.5 (–4.4, 7.7)	0.63	0.41
Traffic density—within 300 m (per 10-fold change in VKT/km^2^/day)^*b*^	–1.9 (–4.9, 1.2)	0.22	0.41	–2.2 (–5.8, 1.5)	0.24	0.42
Dietary cadmium intake (perµg/day)^*f*^	–0.1 (–1.4, 1.4)	0.64	0.42	–0.9 (–2.5, 0.7)	0.16	0.44
*R*^2^, coefficient of determination. ^***a***^Percent change=[exp(β)–1]×100.^***b***^Questionnaire or residence from first urine collection in 2000.^***c***^Questionnaire from follow-up in 1997.^***d***^Among never-smokers responding to the 1997 questionnaire about exposure to secondhand smoke (*n*=171), calculated by multiplying duration of exposure in years by intensity (1=a little smoky; 2,fairly smoky; 3,very smoky).^***e***^Total live and still births from questionnaire at cohort enrollment in 1995–1996.^***f***^Dietary cadmium intake estimated from food frequency questionnaire from 2000 combined with cadmium levels measured in food items from the Total Diet Study (FDA 2006).						

Models with creatinine-adjusted U-Cd or 24-hr U-Cd output as the dependent variable produced parameter estimates similar to those for U-Cd concentration (results not shown). Cross-validation showed that the models were not over fit, with the same independent variables significant in each subset of the data, similar regression coefficients (± 10%) and overall adjusted *R*^2^ values (40–46%).

## Discussion

In this analysis, we observed only a moderate level of within-person correlation for repeated measures of U-Cd concentrations (unadjusted ICC = 0.50; creatinine-adjusted ICC = 0.42) from 24-hr samples collected 3–9 months apart, suggesting that a single measurement does not accurately represent lifetime Cd body burden. This result from repeat 24-hr urine samples is within the range of correlations (*r*⊇=⊇0.4–0.6) observed from the few studies that measured U-Cd in repeat morning void samples (Ikeda et al. 2006; Mason et al. 1998; Yamagami et al. 2008). For an epidemiologic study of the effect of Cd where a single measurement of U-Cd is used to characterize exposure, our observed variance ratio (within- to between-person variance) of one for repeated samples leads to exposure misclassification with an estimated attenuation bias of approximately 50%, such that a “true” odds ratio of 2.0 would be reduced to an observed value of 1.4. Creatinine-adjusted U-Cd levels in this study (GM = 0.38 µg/g) were nearly identical to estimates from other studies in the United States in women of similar age (GM = 0.28–0.36 µg/g) that reported associations between U-Cd and cancer mortality (Adams et al. 2012), breast cancer incidence (McElroy et al. 2006), and cardiovascular mortality (Menke et al. 2009).

In this population of California women without occupational exposure to Cd and a very low prevalence of current smoking (3%), we identified several factors that predicted U-Cd concentrations. Age and lifetime pack-years of smoking were positively associated with U-Cd, consistent with previous studies (Adams et al. 2011; Ikeda et al. 2005; McElroy et al. 2007b; Richter et al. 2009). The association with age, however, may be due to age-related changes in renal physiology such as lower Cd excretion among older individuals due to reduced tubular reabsorption capacity (Bernard 2004; Järup and Åkesson 2009; Vahter et al. 2004) as well as lower absorption of Cd in older women due to postmenopausal increases in serum ferritin (Jian et al. 2009; Milman et al. 1992). Each pack-year of smoking was associated with a 1% increase in U-Cd concentrations in our study population of women who were 31–84 years of age, compared with a 2% increase estimated for a population of premenopausal women who were 40–45 years of age (Adams et al. 2011). Lifetime intensity of passive smoke exposure was also associated with U-Cd among never-smokers in our population. One study observed a significant correlation (*r* = 0.5, *p* = 0.02) between urinary cotinine and U-Cd among 23 children from Sweden (Willers et al. 2005), whereas a study of 254 women in Wisconsin who were 30–69 years of age found no association between U-Cd and self-reported recent passive smoke exposure duration or number of locations where women were exposed, although no measure of smoke intensity was included (McElroy et al. 2007b).

We observed a weak negative relationship between parity and U-Cd concentration, a finding consistent with a recent study of premenopausal women (Adams et al. 2011). Other studies observed a positive association between U-Cd and parity that the study authors attributed to potential iron deficiency during pregnancy resulting in increased absorption of Cd (Åkesson et al. 2002; McElroy et al. 2007a). Average daily alcohol consumption was inversely associated with U-Cd in our study population; this contradicts previous studies that reported no association with any alcohol consumption compared to none (Gil et al. 2011) and ordinal variables similar to our measure of alcohol consumption (McElroy et al. 2007a; Peters et al. 2010). Consistent with previous studies, body surface area, a measure of muscle mass, was inversely associated with U-Cd in bivariate models (Dhooge et al. 2010; McElroy et al. 2007a; Suwazono et al. 2005); however, neither body surface area nor BMI, a measure of adiposity, were associated with U-Cd in adjusted models (data not shown).

Studies of populations consuming food contaminated with Cd have observed positive associations between dietary Cd intake and U-Cd levels (Ikeda et al. 2006; Yamagami et al. 2008) as have several other studies in low-exposure populations (Choudhury et al. 2001; Julin et al. 2011; Shimbo et al. 2000). However, consistent with our findings, other studies of women with low Cd exposure have observed no association between U-Cd levels and either dietary Cd intake (Vahter et al. 1996) or the consumption of specific food items (McElroy et al. 2007a). In a study of nonsmoking women, Adams et al. (2011) observed an association between U-Cd and usual consumption of tofu and cooked cereals. The Total Diet Study, from which we obtained our estimates of dietary Cd in the foods that participants reported consuming, did not include Cd levels in tofu or other soy products. However, tofu was not largely consumed in our population based on FFQs. While some studies measured Cd in duplicate food samples (Julin et al. 2011; Shimbo et al 2000; Vahter et al. 1996) and others relied on FFQs and Total Diet Study data (Choudhury et al. 2001; and the present study), no clear pattern between methodologies and results was apparent. Variation in Cd absorption related to iron stores is another possible explanation for the mixed findings between dietary and U-Cd (McElroy et al. 2007a; Vahter et al. 1996). In addition, Cd levels in food are dependent on soil levels as evidenced by a study in Japan that showed a two-fold variation in Cd levels in foods grown in different locations (Shimbo et al. 2000).

We did not observe associations between estimated Cd exposures from environmental sources and U-Cd in multivariate models. Our study area, which included urban, suburban, and rural regions of the state, was not known to have Cd contamination and had relatively low Cd emissions compared to areas of California with a greater concentration of industrial sources. Furthermore, the ambient levels of Cd observed in the study area are considered to be low and not thought to be a major source of exposure to the general population (ATSDR 1999). Our GIS-derived exposure estimates were based on residential location in 2000 only, and did not account for time spent in other locations (e.g., workplace), wind direction, or meteorology. Nonetheless, the amount of variance in U-Cd concentrations explained by our mixed-effects regression models (*R*^2^ = 42–44%) is similar to that observed in a study (*R*^2^ = 40%) of nonoccupationally exposed women that used a single measure of creatinine-adjusted U-Cd (McElroy et al. 2007a).

Limitations of this analysis include the relatively low and limited range of U-Cd levels and low levels of estimated Cd exposure from smoking and environmental sources among our study participants. Our estimates of potential environmental exposure were based on a single year of data and may not accurately reflect historical cumulative exposure through air. Urine samples were stored for about 9 years at –70°C and were not collected specifically for U-Cd analysis; therefore, potential contamination of the urine collection containers that could contribute to the observed within- and between-person variation in measured concentrations could not be ruled out (McElroy et al. 2007a). Other than tobacco smoke, we were unable to identify sources of exposure that were statistically significant predictors of U-Cd levels. We did not measure our participants’ iron status, which can be an important factor influencing gastrointestinal uptake of Cd (Åkesson et al. 2002; Gallagher et al. 2011; Julin et al. 2011; Satarug et al. 2010). We also did not have any information on renal function such as measures of glomerular filtration that were positively and paradoxically associated with U-Cd in recent studies, suggesting a potential reverse causality (Chaumont et al. 2012; Weaver et al. 2011a, 2011b). The strengths of the present study include use of 24-hr urine samples (Akerstrom et al. 2012); the ability to evaluate within-person variation in U-Cd levels for about half of the study subjects; comprehensive questionnaire data related to dietary, reproductive, and lifestyle factors; and the estimation of Cd exposure from outdoor sources.

## Conclusions

Our results suggest that U-Cd levels increase with age and exposure to tobacco smoke and that a single measurement of 24-hr U-Cd does not accurately reflect medium to long-term (i.e., 6–9 month average) body burden.
